# Quinoxaline Derivatives as Antiviral Agents: A Systematic Review

**DOI:** 10.3390/molecules25122784

**Published:** 2020-06-16

**Authors:** Marc Montana, Vincent Montero, Omar Khoumeri, Patrice Vanelle

**Affiliations:** 1 Laboratoire de Pharmaco-Chimie Radicalaire, Institut de Chimie Radicalaire ICR, Aix Marseille Univ, CNRS, UMR 7273, 13005 Marseille, France; marc.montana@univ-amu.fr (M.M.); vincent.montero@univ-amu.fr (V.M.); omar.khoumeri@univ-amu.fr (O.K.); 2 Oncopharma, Hôpital Nord, Assistance Publique-Hôpitaux de Marseille (AP-HM), 13005 Marseille, France; 3 Service central de la qualité et de l’information pharmaceutiques (SCQIP), Assistance Publique-Hôpitaux de Marseille (AP-HM), 13005 Marseille, France

**Keywords:** quinoxaline, antiviral, SAR, biological applications, chemistry

## Abstract

Background: In recent decades, several viruses have jumped from animals to humans, triggering sizable outbreaks. The current unprecedent outbreak SARS-COV-2 is prompting a search for new cost-effective therapies to combat this deadly pathogen. Suitably functionalized polysubstituted quinoxalines show very interesting biological properties (antiviral, anticancer, and antileishmanial), ensuring them a bright future in medicinal chemistry. Objectives: Focusing on the promising development of new quinoxaline derivatives as antiviral drugs, this review forms part of our program on the anti-infectious activity of quinoxaline derivatives. Methods: Study compiles and discusses recently published studies concerning the therapeutic potential of the antiviral activity of quinoxaline derivatives, covering the literature between 2010 and 2020. Results: A final total of 20 studies included in this review. Conclusions: This review points to a growing interest in the development of compounds bearing a quinoxaline moiety for antiviral treatment. This promising moiety with different molecular targets warrants further investigation, which may well yield even more encouraging results regarding this scaffold.

## 1. Introduction

Humans have a long history of viral infections. For some viral diseases, vaccines and antiviral drugs have made it possible to prevent infections or have helped those infected to recover. Recent antiviral drug development has led to the discovery of effective new treatments to control Human Immunodeficiency Virus (HIV) and Hepatitis C virus (HCV) infections [1]. However, several viruses have jumped from animals to humans triggering sizable outbreaks. One example is the 2014–2016 outbreak of Ebola in West Africa, which resulted in over 28,000 infected patients and was responsible for over 11,000 deaths [2], making it the most lethal member of the Ebola family. The SARS-COV-2 outbreak worldwide continues to pose a serious threat to public health, with no reliable treatment yet available. In addition, the last decade has seen the development of only a few novel antivirals as remdesivir and favipiravir, initially used respectively as Ebola and influenzae treatment and proposed for repurposing in the SARS-CoV-2 outbreak, or sofosbuvir and daclatasvir, which have dramatically changed the prognosis in HCV infection [3,4]. However, this unprecedented SARS-CoV-2 crisis underlines the urgency of developing new cost-effective therapies to combat the deadly pathogen.

Nitrogen-containing heterocycles are promising compounds for the development of new drugs or novel potential lead molecules [5,6,7,8]. The quinoxaline scaffold, a bioisoster of quinoline and naphthalene, is one of the heterocycles currently attracting attention.

Quinoxaline, formed by the fusion of benzene and pyrazine rings, is a white crystalline powder whose melting point is 29–30 °C and whose molecular formula is C_8_H_6_N_2_ [9,10]. Its synthesis has been intensively studied in the past. The classic method of quinoxaline preparation is to condense o-phenylenediamine with a dicarbonyl compound. This procedure requires high temperatures, a strong acid catalyst, and long reaction times [11,12]. Other strategies described for the synthesis of quinoxaline derivatives involve condensation of 1,2-diamines with α-diketones [13], 1,4-addition of 1,2-diamines to diazenylbutenes [14], cyclization–oxidation of phenacyl bromides [15], and oxidative coupling of epoxides with ene-1,2-diamines [16]. There are also several green synthetic methods, like using recyclable catalysts [17], one-pot synthesis [18], microwave-assisted synthesis [19,20], and reactions in aqueous medium [21].

**Figure 1:**
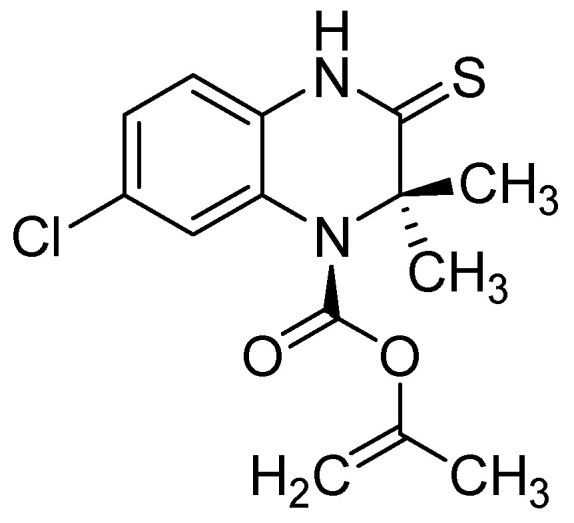
Chemical structure of S-2720.

Suitably functionalized polysubstituted quinoxalines show very interesting biological properties (antiviral [22], anticancer [23], and antileishmanial [24]), ensuring them a bright future in medicinal chemistry [11,25]. Many drug candidates bearing quinoxaline core structures have been identified, such as S-2720 (Figure 1), found to be a very potent inhibitor of HIV-1 reverse transcriptase [26].

This review investigates the new quinoxaline derivatives that are showing promise as antiviral drugs as part of our program focused on the anti-infectious activity of quinoxaline derivatives. It compiles and discusses recently published studies concerning the therapeutic potential of the antiviral activity of quinoxaline derivatives, covering the literature between 2010 and 2020.

## 2. Methods

### 2.1. Background Definition

The search method employed in this systematic review was to select studies that evaluated the biological activity and mechanism of action of quinoxaline derivatives.

### 2.2. Data Sources and Searches

Three different databases were used to conduct a comprehensive survey: MEDLINE/PubMed (National Library of Medicine—www.ncbi.nlm.nih.gov/pubmed), Web of Science (Thomson Reuters Scientific—www.webofknowledge.com/), and Science Direct (Elsevier www.sciencedirect.com). The search terms “quinoxaline” and “antiviral” were chosen so as to detect everything published about quinoxaline before applying exclusion criteria. Searches were conducted using the limit dates of 1 January 2010 and 1 May 2020.

### 2.3. Study Selection

The review was performed in three main stages by three independent reviewers. In the first stage, articles’ titles and abstracts were assessed according to the eligibility criteria (Table 1). In the second stage, duplicated articles were deleted. Finally, the authors read each selected full text and eliminated articles fitting the exclusion criteria. During this final stage, articles found in the reference lists of selected manuscripts, but which had not been listed under the search terms in the databases, were added.

### 2.4. Data Extraction Process

The following information was extracted from all the selected studies: type of study, biological matrix used, compound structure and nomenclature, and main conclusions.

## 3. Results

The database search identified 216 records. After the first evaluation phase (title/abstract), 175 records were excluded. Eight repeated files were discarded, leaving 33 articles.

No other paper was added from the reference lists of the identified studies (which had not been found in the initial search). A second phase was therefore conducted with a total of 33 articles.

**Figure 2:**
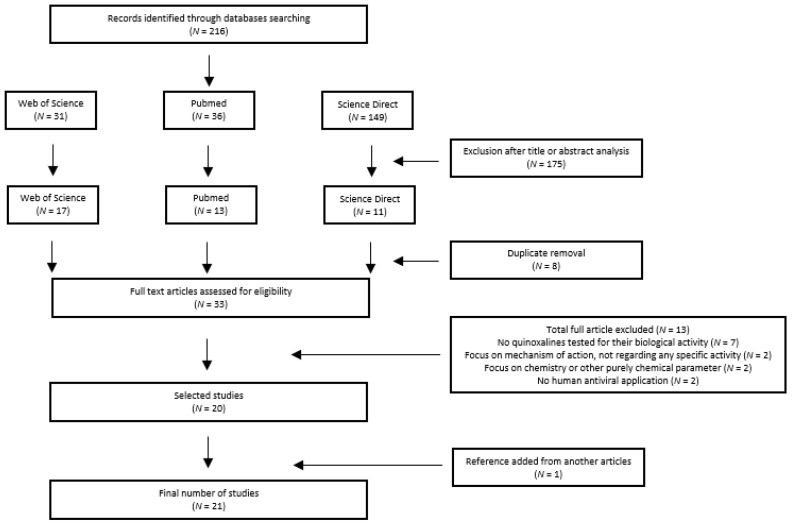
Flow diagram of study selection adapted from PRISMA [27].

After the full-text reading, 13 articles were excluded and one was included, leaving a final total of 21 studies included in this review. This process is illustrated by a flow diagram in Figure 2.

After critical reading, articles were divided into categories according to the activity assessed in each study (Table 2). A manuscript could be assigned to more than one category if a single study evaluated more than one type of antiviral activity.

## 4. Discussion

### 4.1. Quinoxaline Derivatives Active against DNA Viruses

DNA viruses have DNA genomes that are replicated by either host or virally encoded DNA polymerases. DNA viruses with a large genome, particularly the families of *Herpesviridae* and *Poxviridae*, encode a number of proteins that counter host defenses. [48] Double-stranded DNA viruses can be subdivided into three groups: (1) those with a small size DNA genome (<10 kb), such as polyomaviruses and papillomaviruses; (2) those with a medium-sized DNA genome (ca., 35 kb), such as adenoviruses; and (3) those with a large DNA genome (ca., 130–250 kb), such as herpesviruses and poxviruses [49].

#### 4.1.1. Quinoxaline Derivatives Active against *Herpesviridae*

The *Herpesviridae* are a ubiquitous worldwide family responsible for viral infections. Among its members frequently encountered are, herpes simplex viruses (HSV-1 and HSV-2), human cytomegalovirus (HCMV), and Epstein Barr virus (EBV) [28,50,51]. Usually, these infections remain latent and patients are often asymptomatic, particularly immunocompetent populations. However, in immunocompromised patients especially (e.g., AIDS, cancer, etc.), there can be clinical symptoms such as meningitidis or pneumoniae leading to death. Treatments against HSV or HCMV like ganciclovir, valganciclovir, foscarnet, or cidofovir, are currently available but they are limited by toxicity and/or poor oral bioavailability [28,52,53]. In addition, drug resistance is emerging. Hence there is a need to identify improved agents that circumvent one or more of these problems.

**Figure 3:**
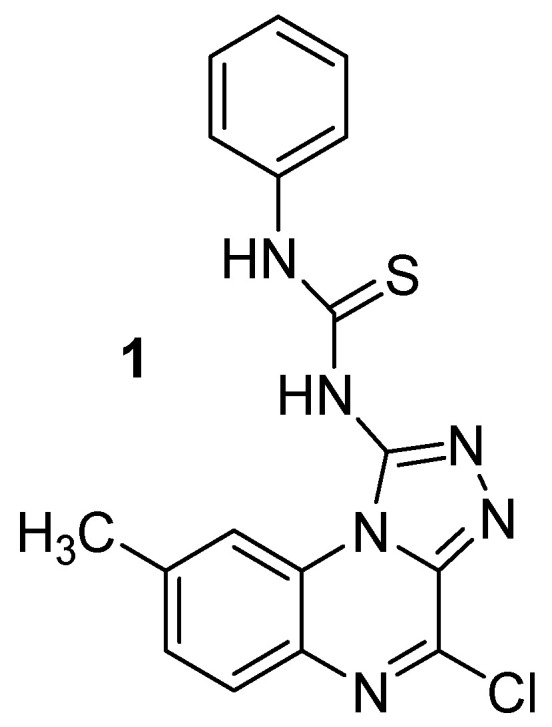
Chemical structure of 1-(4-chloro-8-methyl[1,2,4]triazolo[4,3*a*]quinoxaline-1-yl)-3-phenyl thiourea **1**.

In 2012, a series of new [1,2,4]triazolo[4,3-*a*]quinoxaline derivatives and their pyrimido-quinoxaline isosters were synthesized and evaluated as potential antiviral agents. Twenty-two novel compounds were obtained. Among them, 1-(4-chloro-8-methyl[1,2,4]triazolo[4,3*a*]quinoxaline-1-yl)-3-phenyl thiourea **1** showed the highest antiviral activity in a plaque-reduction assay against Herpes simplex virus grown on Vero African monkey kidney cells, reducing the number of plaques by 25% at 20 µg/mL (Figure 3). Nine other compounds reduced the number of plaques by less than 25% at 80 µg/mL, leading the authors to the conclusion that the thiourea moiety may be responsible for antiviral activity and highlighting the importance of the moieties selected in developing antiviral activity [29]. However, antiviral activity remained disappointing compared to positive control aphidicolin, which reduced the number of plaques by 100% at 5 µg/mL, even though compound **1** showed lower cytotoxicity.

**Figure 4:**
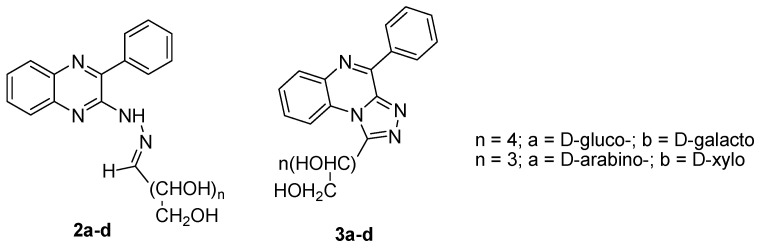
Chemical structure of new series of aldehydo-sugar-N-(3-phenylquinoxalin-2-yl)hydrazones and their acyclic C-nucleoside analogues, 1-(4-phenyl-[1,2,4]triazolo[4,3-*a*]quinoxalin-1-yl)alditols.

The synthesis of four new aldehydo-sugar-N-(3-phenylquinoxalin-2-yl)hydrazones **2a-d** and their acyclic C-nucleoside analogues, 1-(4-phenyl-[1,2,4]triazolo[4,3-*a*]quinoxalin-1-yl)alditols **3a-d** (Figure 4) indicated that these compounds exhibited very weak antiviral activity against HSV-1 in a plaque reduction infectivity assay in comparison to aphidicolin taken as reference [30].

Recently, nine novel quinoxaline derivatives were synthetized via different nucleophilic reactions using ethyl (6,7-dimethyl-2-oxo-3,4-dihydroquinoxalin-3-yl)acetate **4** and ethyl (6-methyl-2-oxo-3,4-dihydroquinoxalin-3-yl)acetate **5**, 3-methylquinoxalin-2(1*H*)-one, and 1,4-dihydroquinoxaline-2,3-dione as precursors [31] (Table 3). When their antiviral activity against HCMV was compared to the standard drug ganciclovir (EC_50_ = 0.059 µM), two derivatives demonstrated higher activity, each with EC_50_ < 0.05 µM. Notably, the toxicity of **4** and **8** (CC_50_ = 108.47 and >150 µM, respectively) was comparable to the reference drug (CC_50_ >150 µM) and compound **6** showed the poorest safety profile with CC_50_ = 2.34 µM.

**Figure 5:**
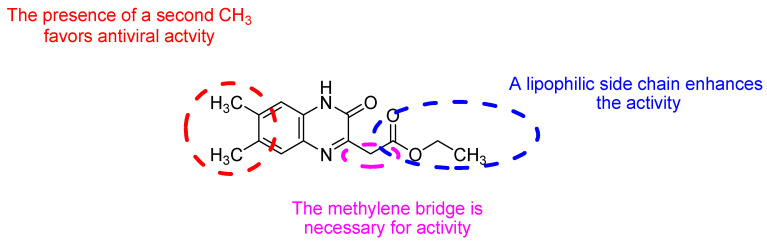
Structure–activity relationship of novel quinoxaline derivatives with anti HCMV activity.

Four other compounds showed promising antiviral activity. Antiviral activity was observed to depend on varying chemical characteristics, like the presence of a dimethylquinoxalinyl methylene nucleus as a common structural feature and the presence of a lipophilic ester function (Figure 5).

**Figure 6:**
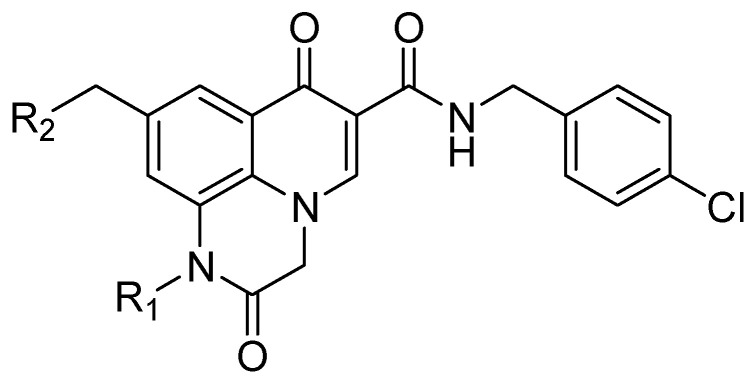
General structure of 2-aryl-2-hydroxy ethylamine substituted 1*H*,7*H*-pyrido[1,2,3-*de.*]quinoxaline-6-carboxamides synthetized.

In addition, several quinoxaline derivatives are of current interest as anti HCMV agents [28]. Some 2-aryl-2-hydroxy ethylamine substituted 1*H*,7*H*-pyrido[1,2,3-*de*]quinoxaline-6-carboxamides were synthetized and tested (Figure 6).

In this study, the pyridoquinoxaline nucleus proved to be a useful nucleus, as some of the synthetized compounds showed a favorable profile for established drugs like as ganciclovir, acyclovir, foscarnet, and aphidicolin [28] (Table 4),

The morpholinomethyl side chain afforded good levels of both enzymatic and antiviral activity whereas the benzofuran moiety resulted in extremely potent enzymatic and antiviral activity. However, despite excellent biological activity, the calculated log P of compound **10** proves the need to continue pharmacomodulation efforts aimed at improving its hydrosolubility.

**Figure 7:**
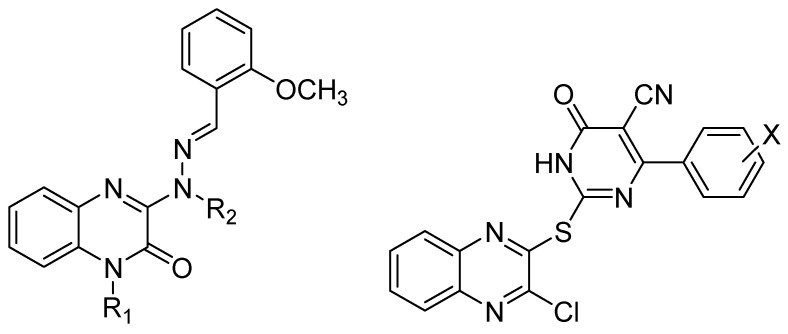
General structures of 3-aminoquinoxalin-2(1*H*)-one derivatives and derivatives with pyrimidine ring linked to quinoxaline through sulfur exhibiting anti EBV antigen activation.

Finally, EBV is a very common virus that can increase the risk of developing certain rare cancers. The malignant transformation of normal human epithelial cells results from exposure to EBV and that transformation is dependent on the presence of phorbol esters, which stimulate cell proliferation through rapid activation of protein kinase C [32,54]. Novel 3-amioquinoxalin-2(1*H*)-one derivatives and derivatives with pyrimidine ring linked to quinoxaline through sulfur (Figure 7) exhibited properties against EBV antigen activation.

On a series of 22 original compounds, six derivatives demonstrated stronger inhibitory effect on EBV than oleanolic acid as reference, without showing any cytotoxicity. The structure–activity relationship proved that disubstitution with alkyl groups on both nitrogen of hydrazine and quinoxaline was crucial for activity especially for the allyl group. This high activity could result from a hydrophobic interaction between the alkyl group and the hydrophobic region of the binding site of the receptor. The presence of a methoxy group on the phenyl group and substitution with a pyrimidine nucleus linked to quinoxaline through sulfur were also conducive to activity [32].

#### 4.1.2. Quinoxaline Derivatives Active against *Poxviridae*

The *Poxviridae* family include 38 viruses that can infect a wide range of hosts, including mammals, birds, reptiles, and insects [55]. The causative agent of Smallpox and Molluscum contagiosum, two human specific diseases, belongs to the poxviruses. Although variola was globally eradicated, Molluscum contagiosum results from a usually benign infection with mild skin disease characterized by lesions that may appear anywhere on the body. Within 6–12 months, Molluscum contagiosum typically resolves without scarring, but may take as long as many years in some people with weakened immune systems [56].

**Figure 8:**
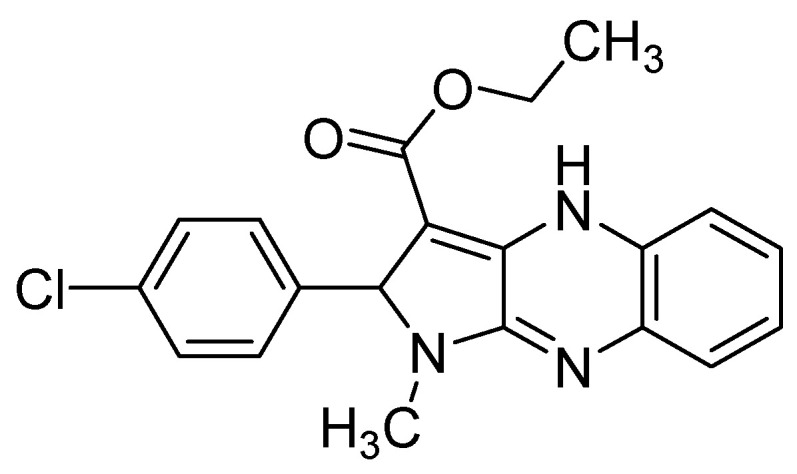
Structure of the lead compound **11** for poxvirus inhibition.

In a series of nine new halophenyl pyrrolo[2,3-*b*]quinoxaline derivatives, none of the compounds proved inhibitory at subtoxic concentration except ethyl 2-(4-chlorophenyl)-1-methyl-2,4-dihydro-1*H*-pyrrolo-[2,3-*b*]quinoxaline **11** (Figure 8), which inhibited the vaccinia virus and was considered as a potential lead compound for poxvirus inhibition, with an EC_50_ value of 2 µM in HEL cell cultures and moderate antiproliferative activity (CC_50_ >20 µM) [33].

#### 4.1.3. Quinoxaline Derivatives Active against *Hepadnaviridae*

Hepatitis B virus (HBV) is a member of the *Hepadnaviridae* family. This virus can cause liver infections that lead to various hepatic diseases such as hepatitis, cirrhosis, and hepatic cancer. A new class of 3-(1′,2′-dihydroxyeth-1′-yl)-1-phenylpyrazolo[3,4-b]quinoxaline demonstrated encouraging anti-hepatitis B activity at 100 µM, but the five most potent compounds were associated with high cytotoxicity (cytotoxicity >30% at 100 µM) [34].

### 4.2. Quinoxaline Derivatives Active against ARN Viruses

Human disease-causing RNA viruses include Orthomyxoviruses, Hepatitis C Virus (HCV), Ebola disease, SARS, and retroviruses including adult human T-cell lymphotropic virus type 1 (HTLV-1) and human immunodeficiency virus (HIV). RNA viruses have RNA as genetic material, either single-stranded RNA or double-stranded RNA. Viruses may exploit the presence of RNA-dependent RNA polymerases for replication of their genomes or, in retroviruses, reverse transcriptase produces viral DNA, which can be integrated into the host DNA under its integrase function [57].

#### 4.2.1. Quinoxaline Derivatives Active against *Picornaviridae*

Enteroviruses, which include coxsackievirus A and B, belong to *Picornaviridae*, a single-stranded RNA virus family. They are implicated in various diseases, with a wide range of symptoms; and exceptionally, coxsakieviruses can be responsible for more severe diseases, such as flaccid paralysis myocarditis, pericarditis, encephalitis, or systemic neonatal disease [35,58,59]. To date, there are conventional treatments or vaccines against coxsackieviruses, which cause acute or chronic disease in infants, children, and immunocompromised persons.

**Figure 9:**
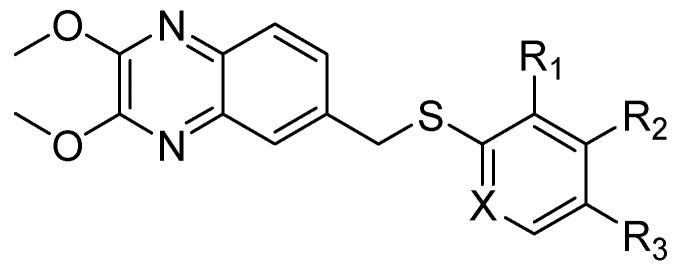
Chemical structure of quinoxaline derivatives.

In order to develop more effective antivirals, 14 new quinoxaline derivatives (Figure 9) were synthetized and tested against a panel of viruses for which the efficacy of therapeutic agents was unsatisfactory [35].

Among these new quinoxalines, ethyl 4-(((2,3-dimethoxyquinoxalin-6-yl)methyl)thio)benzoate **11**, 4-(((2,3-dimethoxyquinoxalin-6-yl)methyl)thio)benzoic acid **12** and ethyl 6-(((2,3-dimethoxyquinoxalin-6-yl)methyl)thio)nicotinate **13** displayed remarkable activity against coxsackievirus B5 (CBV5), with an EC_50_ = 0.09 µM, 0.06 µM, and 0.3 µM, respectively (Table 5). The absence of cytotoxicity towards the Vero-76 cells of compound **11** led to further experimental/in silico investigation aimed at determining its mechanism of action. These investigations demonstrated that compound **11** inhibits CBV5 by targeting the early events of attachment, entry or uncoating, as it can favorably insert into a hydrophobic pocket on the VP1 chain of the capsid protomer implicated in the protein conformational changes during infection of the host cell.

#### 4.2.2. Quinoxaline Derivatives Active against *Orthomyxoviridae*

Influenza viruses can cause contagious respiratory disease in humans and are responsible every year for flu pandemics.

Based on the planar polyaromatic system (chromophore), quinoxaline derivatives are good candidates to combat influenza viruses because of their potential to target the NS1 protein, a highly conserved influenza virus encoded protein. Since the N-terminal domain of the NS1A protein results in a six-helical chain fold with a deep cavity at the center of the double-stranded RNA-binding surface, a small molecule could fit into the cavity and block virus replication [36,60,61,62]. 2,3,6-substitued quinoxaline has also yielded compounds identified as having valuable antiviral activity, particularly under bis-2-furyl substitution. Maintaining bis-2-furyl substitution, a novel series of quinoxaline derivatives was synthetized to determine the influence of substitution at position 6. Two derivatives, one with 3-methoxyphenyl group and one with 2-furyl at position 6, showed good activity with an IC_50_ of 6.2 and 3.5 µM, respectively (Table 6). RNA intercalation experiments showed that both compounds could bind to the NS1A RNA-binding domain, demonstrating the antiviral potential of these quinoxaline derivatives [36].

#### 4.2.3. Quinoxaline Derivatives Active against *Filoviridae*

Ebola and Marburg belong to the *Filoviridae* family of single-stranded RNA viruses. These viruses were responsible for the 2014–2015 outbreak of hemorrhagic fever in Western Africa, resulting in a total of 28,616 infected people, including 11,310 deaths, for a case-fatality rate of 40%. There is currently no antiviral drug licensed by the U.S. Food and Drug Administration (FDA) to treat Ebola infection; however, four drugs called ZMapp remdesivir, Mab114, and REGN-EB3, are under investigation, as each has reduced the risk of death from Ebola [63,64]. Actually, the outbreak is not yet over, with new cases identified in the Democratic Republic of Congo, and the need for antiviral candidates remains strong.

**Figure 10:**
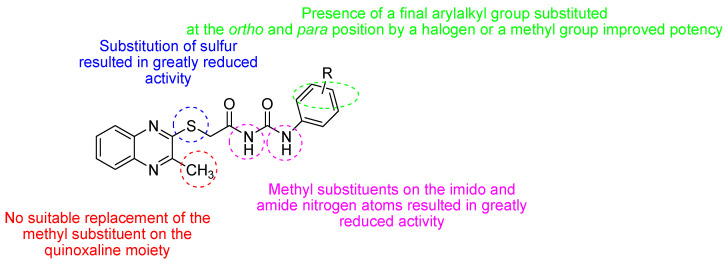
Structure–activity relationship of novel series of quinoxaline-2-mercapto-acetyl-urea analogues.

A critical virus–host interaction required for virus egress and dissemination involves late-budding domains containing PPxY motifs, which are highly conserved in the matrix protein of a large number of RNA viruses. Targeting this interaction, a novel series of quinoxaline-2-mercapto-acetyl-urea analogues (Figure 10) were synthetized and evaluated for their ability to inhibit viral egress of Marburg and Ebola in VP40 VLP budding assay in HEK293T cells [2]. Among them, four compounds demonstrated strong RNA viral egress inhibition potential.

#### 4.2.4. Quinoxaline Derivatives Active against *Flaviviridae*

HCV is responsible for both acute and chronic hepatitis, ranging in severity from a mild illness lasting a few weeks to a serious, lifelong illness. Globally, an estimated 71 million people have chronic HCV infection and in 2016, approximately 399,000 people died from HCV [65]. Antiviral drugs can cure more than 95% of HCV patients, but access to treatment is poor due to its high cost, which is why research is ongoing.

**Figure 11:**
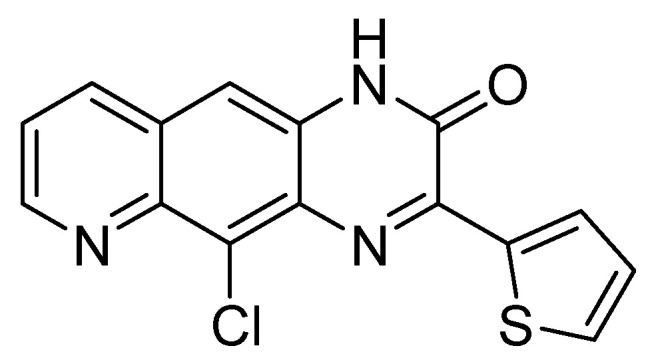
5-chloro-3-(thiophen-2-yl)pyrido[2,3-*g*]quinoxaline-2(1*H*)-one **16.**

Several quinoxaline derivatives were evaluated for their anti-HCV potential. Even though novel quinoxaline derivatives synthetized using ethyl (6,7-dimethyl-2-oxo-3,4-dihydroquinoxalin-3-yl)acetate **4** and ethyl (6-methyl-2-oxo-3,4-dihydroquinoxalin-3-yl)acetate **5**, 3-methylquinoxalin-2(1*H*)-one, and 1,4-dihydroquinoxaline-2,3-dione as precursors failed to demonstrate any activity against HCV [31], in pyrido[2,3-*g*]quinoxalinone series, 5-chloro-3-(thiophen-2-yl)pyrido[2,3-*g*]quinoxaline-2(1*H*)-one **16** (Figure 11) was able to inhibit HCV replication in a subgenomic replication assay with EC_50_ = 7.5 ± 0.5 µM. However, it was also cytotoxic for GS4.1 cells (CC_50_ = 21 ± 20 µM) [37,38,55].

**Figure 12:**
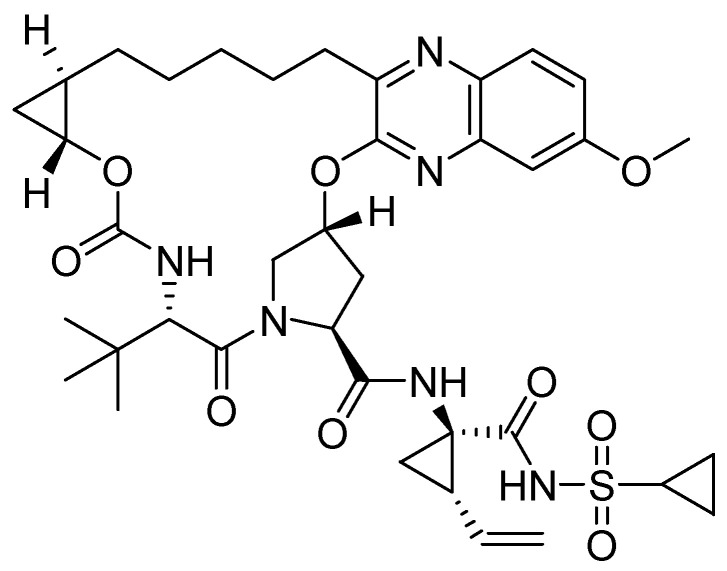
Chemical structure of grazoprevir.

However, grazoprevir **17**, a novel P2-P4 quinoxaline macrocyclic NS3/4a protease inhibitor with broad activity across genotypes and resistant variants, is currently approved for the treatment for HCV [39] (Figure 12).

The structure–activity relationship shows that grazoprevir’s efficacy derives from lipophilic interaction at P2 position in addition to a contribution from the P2-P4 constraint [66]. The P2 quinoxaline moiety largely avoids direct interaction with residues Arg-155 and Asp-168, the two most common resistance-associated residues, but interacts with the catalytic His-57 and Asp-81, which explains its activity against most HCV genotypes and resistant variants [67]. Modeling studies showed that in patients who failed to achieve sustained virologic response with simeprevir, grazoprevir was efficacious because of a strong direct cation–quinoxaline interaction with the Lys-155 side chain of double substitution R155K/D168A [40]. More recently, eliminating the P2-P4 moiety was considered aiming at conformational flexibility and exploration of diverse quinoxalines at position P2 in order to improve potency and resistance profile. The structure–activity relationship indicated that a small hydrophobic substituent at position 3 of P2 quinoxaline effectively maintains activity against resistant variants, as derivatives with a larger group at position 3 of P2 quinoxaline shift out of the binding site [41,42]. Further investigations involved replacement of the quinoxaline moiety by a quinoline scaffold, leading to interesting analogues [68].

#### 4.2.5. Quinoxaline Derivatives Active against *Rhabdoviridae*

**Figure 13:**
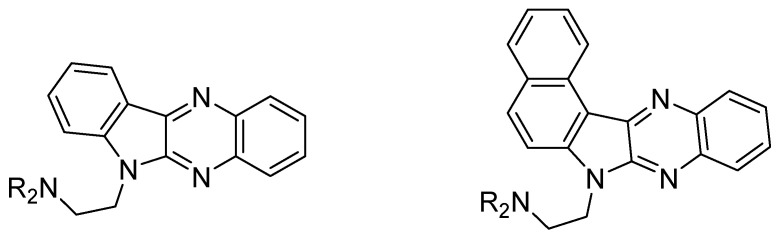
General structure of synthetized indoloquinoxalines and benzoindoloquinoxalines.

Vesicular stomatis virus (VSV) is a virus in the family *Rhabdoviridae*. This virus is zoonotic and in infected humans leads to a flu-like illness characterized by fever, headache, myalgia, weakness, and occasionally vesicular lesions of the mouth [69]. As VSV infection results in a short 3–5-day illness, no specific treatment is available and VSV is commonly used as a laboratory virus to study the properties of viruses. Indoloquinoxaline derivatives and their benzoindoloquinoxalines were synthetized and assessed for their anti-VSV activity, interferon-inducing ability, and cytotoxicity (Figure 13). Anti-viral activity was significantly reduced with annulation of benzene ring in indoloquinoxaline derivatives [43,44].

As these indoloquinoxalines were more active antivirals when they were added immediately after virus infection, it was supposed that their antiviral action was first mediated by interferon.

#### 4.2.6. Quinoxaline Derivatives Active against *Retroviridae*

**Figure 14:**
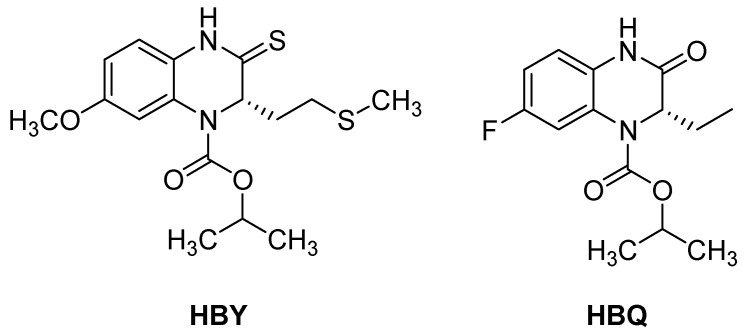
Chemical structure of HBY and HBQ.

While there is no cure for HIV, there are very effective treatments that enable most people with the virus to live a long and healthy life. Combination antiretroviral therapy is required for durable virologic suppression. Reverse transcriptase is one of the most frequent targets for the treatment of HIV infection, since the blockage of this enzyme can stop an essential step in viral replication. However, a growing number of cases of resistant HIV strains and serious adverse events due to the antiretroviral therapy administered have encouraged attempts to develop new HIV agents, more active, less toxic, and with increased tolerability to mutation [70]. Some quinoxaline derivatives like HBY, HBQ, and S-2720 have demonstrated high potency as reverse transcriptase inhibitors (Figure 14).

For these reasons, design, synthesis, and evaluation of new quinoxaline derivatives was investigated. Using a computational approach, 58 quinoxaline compounds were identified, and 25 new quinoxaline and quinoxaline-related compounds were synthetized and evaluated as inhibitors of reverse transcriptase (RT). Chemical features identified as crucial for reverse transcriptase inhibition were the presence of a five- or six-membered aromatic ring and a hydrophilic center that can be nitrogen, oxygen, or sulfur. Six of these derivatives presented the highest inhibitory activity at 100 µM, ranging from 56% to 99% of reverse transcriptase inhibition, and were considered as hit compounds. One compound was a particularly interesting derivative, with values comparable to those of commercial compound nevirapine when used at 10 µM (both showing reverse transcriptase inhibition % = 91) [45] (Table 7).

Compound **19** displayed similar inhibitory activity with nevirapine, with an EC_50_ = 3.1 nM vs. EC_50_ = 6.7 nM respectively and can be considered a promising lead compound [45].

Moreover, this new class of integrase inhibitors proved very effective against HIV, showing a high therapeutic index. Three representatives of this class, raltegravir, elvitegravir, and dolutegravir are currently available. However, for two of them have led to reported cases of resistance, indicating to an urgent need to develop other new effective anti-HIV agents. In a structure-based drug design approach, the quinoxaline scaffold was identified as a core moiety to design potential novel anti-HIV agents. A series of seven new 6-chloro-7-fluoroquinoxaline derivatives with various substituents at position 2 and 3 was also synthetized. Among them, two derivatives **23** and **24** bearing bulky substitution at position 2 and 3 exhibited better activity compared to unsubstituted or less bulky substitutions [46]. In addition, these two compounds revealed no cytotoxicity on VERO cells (Table 8).

**Figure 15:**
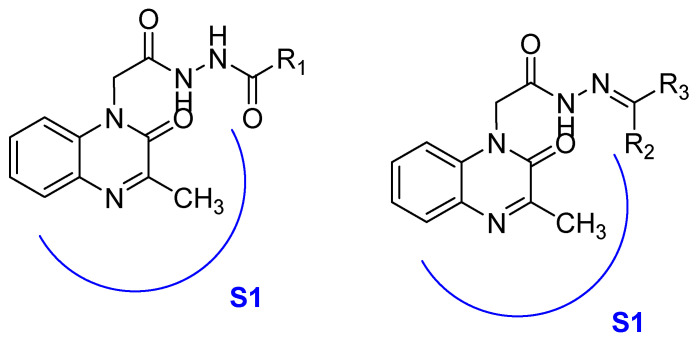
Structure and model of the binding site of new gelatinase inhibitors.

Recently, an approach aimed at dysregulation of gelatinase and pathogenesis of HIV led to the synthesis of two new classes of gelatinase inhibitors bearing a quinoxalinone motif, based on this coplanar scaffold being able to penetrate into the relatively broad S1 binding domain of gelatinase. The acylamide (Series I) and acylhydrazone (Series II) linkage can also act as potent H-bonding acceptor/donor to interact with the active amino acid of the enzyme [47] (Figure 15).

Derivatives in series 1 displayed moderate activity with gelatinase enzymatic inhibition ranging from 34.79 ± 6.3 µM to >500 µM against 5.64 ± 0.6 µM for LY52 taken as reference. The best activity was observed for a *para*-chloro phenyl substituent. In series II, two derivatives **25** and **26** demonstrated similar activity with LY52, probably because the substituents introduced have enough space and the right orientation to guide the compounds to fit into the binding cavity (Table 9). In addition, as compound **26** displayed slightly more potent activity than compound **25**, it was concluded that the phenolic hydroxyl group could provide a more effective hydrogen-bonding interaction, resulting in increased affinity. Substitution with an aliphatic group led to inactive compounds.

## 5. Conclusions

Quinoxaline represent an important class of nitrogen-containing heterocycles with a wide range of potential biological activities. This review points to a growing interest in the development of compounds bearing a quinoxaline moiety for antiviral treatment. Regarding the antiviral activity of quinoxaline derivatives, studies showed that these derivatives represented very encouraging agents for investigators as they exhibit some activity against a large number of different viruses. Future investigations of this moiety requiring analysis of structure–activity relationships, as well as the mechanisms of action of these compounds could give some more encouraging results and may provide to new useful therapeutics.

## Figures and Tables

**Table 1:** Inclusion and exclusion criteria.

	Parameter	Inclusion	Exclusion
1	Language	English, French	Any other language
2	Type of study	Biological activity, In vitro and/or in vivo studies	Exclusively in silico, articles that focus only on synthesis or other purely chemical parameters
3	Type of publication	Original manuscripts	Reviews, book chapters, posters, table of contents, personal opinions, indexes, conference abstracts, letters
4	Search terms		Merely citing keywords in text
5	Mechanism of action	Articles that evaluate the biological activity of quinoxaline derivatives	Articles that concern non-human or animal viruses

**Table 2:** Activities associated with quinoxaline derivatives based on virus classification.

Activity	Reference
**DNA viruses**	
*Herpesviridae*	[28,29,30,31,32]
*Poxviridae*	[33]
*Hepadnaviridae*	[34]
**RNA viruses**	
*Picornaviridae*	[35]
*Orthomyxoviridae*	[36]
*Filoviridae*	[2]
*Flaviviridae*	[31,37,38,39,40,41,42]
*Rhabdoviridae*	[43,44]
*Retroviridae*	[45,46,47]

**Table 3:** Chemical structure, and EC_50_ values against human cytomegalovirus (HCMV).

Compound	Structure	EC_50_ (µM)
**4**	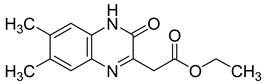	<0.05
**5**	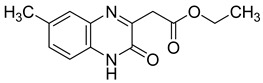	>30
**6**	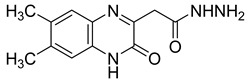	>1.2
**7**	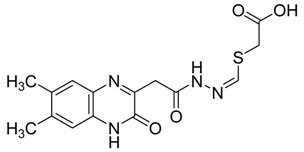	>30
**8**	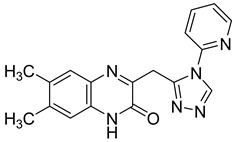	<0.05
Ganciclovir	-	0.059

EC_50_: Compound concentration that reduces viral replication by 50%.

**Table 4:** HCMV pol/pra IC_50_ for lead compounds.

Compound	Structure	HCMV pol Activity IC_50_ (nM)	HCMV pra Activity IC_50_ (nM)
**9**	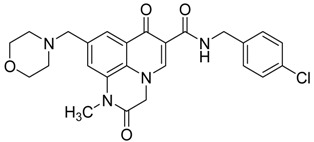	620	100
**10**	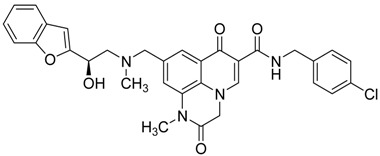	4	1
Ganciclovir		-	1300
Acyclovir		-	>20,000
Foscarnet		2500	-
Aphidicolin	-	487	-

Pol: enzymatic activity; Pra: antiviral activity.

**Table 5:** Chemical structures, activity against coxsackievirus B5 and cytotoxicity.

Compound	Structure	EC_50_ CBV5 (µM)	CC_50_ Vero-76 cells (µM)
**11**	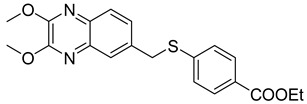	0.09 ± 0.01	>100
**12**	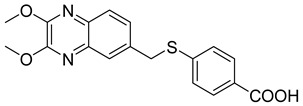	0.06 ± 0.01	>65
**13**	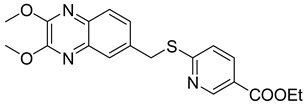	0.3 ± 0.05	>100

EC_50_: Compound concentration that reduces the plaque number of CBV5 by 50%. CC_50_: Concentration of a drug that will kill 50% of cells in uninfected cell culture.

**Table 6:** Structure and activity of 2,3,6-substitued quinoxaline.

Compound	Structure	% Binding at 50 µM	% Intercalation at 50 µM
**14**	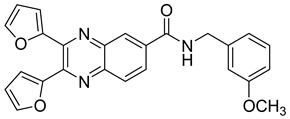	74.0	4.5
**15**	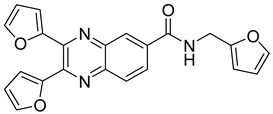	79.5	5.9

**Table 7:** Activity of new potential quinoxaline derivatives as inhibitors of reverse transcriptase.

Compound	Structure	%RT Inhibition		MT2 IC_50_ (µM)	Selectivity Index (SI)
		100 µM	10 µM		
**18**	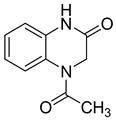	56	7	NE	NE
**19**	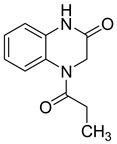	99	91	0.63 (0.53–0.76)	31,798
**20**	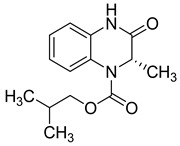	64	37	64 (38–103)	NE
**21**	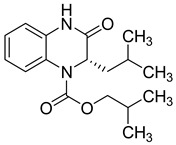	51	22	67 (54–87)	74
**22**	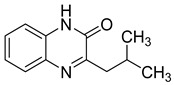	73	15	23 (21–25)	NE
**Nevirapine**	-	98	91	0.13 (0.09–0.17)	14,353

NE: Not evaluated.

**Table 8:** HIV activity and cytotoxicity study of the two most potent new 6-chloro-7-fluoroquinoxaline derivatives.

Compound	Structure	Strain IIIB IC_50_ (µg/mL)	Vero IC_50_ (µg/mL)
**23**	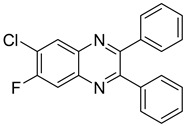	>11.78	>100
**24**	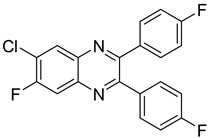	>15.45	>100

**Table 9:** Enzymatic inhibition and cytotoxicity study of tested compounds.

Compound	Structure	Gelatinase IC_50_ (µM)	C8166 CC_50_ (µM)
**25**	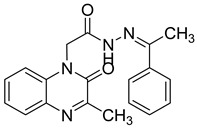	9.39 ± 0.7	>100
**26**	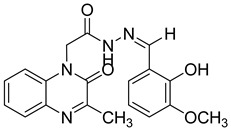	7.17 ± 0.95	>100
**LY52**		5.64 ± 0.6	>100
